# Association of methylenetetrahydrofolate reductase *(MTHFR)* variant *C677T* and risk of carotid atherosclerosis: a cross-sectional analysis of 730 Chinese Han adults in Chongqing

**DOI:** 10.1186/s12872-020-01505-1

**Published:** 2020-05-13

**Authors:** Xulei Peng, Yongli Zhou, Xiaoxing Wu, Xiaolin Wang, Huili Bai, Yongqiang Li, Zhichao Wang, Xuan Chen, Yonghong Wang

**Affiliations:** 1 Department of Neurology, The First Affiliated Hospital of Chongqing Medical University, Chongqing, China; 2 Health Management Department, Chongqing General Hospital, Chongqing, China; 3 Department of Gastrointestinal Surgery, The First Affiliated Hospital of Chongqing Medical University, Chongqing, China; 4 Health Management Center, The First Affiliated Hospital of Chongqing Medical University, Chongqing, China; 5 Department of the Clinical molecular Center, The First Affiliated Hospital of Chongqing Medical University, Chongqing, China; 6 Emergency Department, The Third Affiliated Hospital Of ChongQing Medical University, Chongqing, China

**Keywords:** Methylenetetrahydrofolate reductase, Carotid arterosclerosis, Chinese, Risk factors

## Abstract

**Background:**

Uncertainty still remains on the correlation of methylenetetrahydrofolate reductase (*MTHFR*) variant *C677T* with risk of carotid atherosclerosis (CAS), and there is a lack of reports on *C677T/MTHFR* in the Asian population. The association of *C677T/MTHFR* polymorphisms with CAS in the Chinese Han population in Chongqing was investigated in the present study.

**Methods:**

Subjects (*n =* 730, 214 females and 516 males, Han ethnicity) who provided an informed consent were randomly selected from the general population of Chongqing, China. Polymerase chain reaction-restriction fragment length polymorphism and Sanger sequencing genotyping assays were used to determine the *MTHFR* genotypes. The atherosclerosis index of the intima-media thickness (IMT) was measured by high-resolution ultrasound to evaluate the CAS. Less than 1.0 mm was considered as normal for IMT, 1.0–1.5 mm was considered as thickening, and ≥ 1.5 mm and a local bulge thickened in the lumen was considered as CAS. According to the carotid ultrasonography results, these subjects were divided into two groups: CAS-group (IMT ≥ 1.0 mm) and control group (IMT < 1.0 mm).

**Results:**

The frequency of *C/T* heterozygotes, *T/T* homozygotes genotype was significantly higher in the subjects with CAS (62% vs. 36.9%; 16.2% vs. 9.5%; 47.2% vs. 27.9%, *P* < 0.05), while the frequency of *C/C* homozygotes and *C* allele was significantly lower (21.8% vs. 53.7%; 52.8% vs. 72.1%, *P* < 0.05), when compared to the control group. The risk of CAS was higher for subjects with *C/T* heterozygotes and *T/T* homozygotes (OR = 4.06, 95% CI: 2.76–5.98, *P* < 0.001 and OR = 3.14, 95% CI: 1.73–5.69, *P* < 0.001, respectively), when compared to the subjects with the *C/C* genotype. In the model 1 (*CT + TT* versus *CC*), *C677T/MTHFR* was significantly associated with the prevalence of CAS, and the all adjusted OR values for CAS were 3.87 (95% CI, 2.67 to 5.62) in all, 17.18 (95% CI, 7.27 to 40.49) in women and 2.57 (95% CI, 1.65 to 3.99) in men after adjusting for potential confounding factors.

**Conclusions:**

The present study suggests that a mutation in the methylenetetrahydrofolate reductase gene is a risk factor of CAS in the Chinese Han population.

## Background

Atherosclerosis is a systemic, inflammatory and progressive chronic systemic disease characterized by the affected arteries, carotid IMT thickening, and lipid accumulation. Hence, fibrous tissue hyperplasia and medial calcification eventually leads to the thickening and hardening of the arterial wall, atheromatous plaque formation, and the narrowing of the lumen. The carotid artery is the earliest involved blood vessel in the development of atherosclerosis. Furthermore, some studies have revealed that carotid ultrasound measurement, which is usually carotid IMT, is closely correlated with the severity of extracranial CAS [1].

Homocysteine (Hcy) is a sulfur-containing non-proteinogenic amino acid derived in methionine metabolism. The increased level of Hcy in plasma is hyperhomocysteinemia (HHcy), and there is a clear clinical association between HHcy and both cardiovascular and cerebrovascular disease, as well as diabetic nephropathy [2]. In various studies, HHcy has been considered as a common, independent and alterable risk factor for cardiovascular disease (CVD). Approximately 40% of patients diagnosed with early coronary, cerebrovascular, or peripheral vascular diseases have HHcy [3]. However, it remains uncertain whether Hcy can be used as a marker or causative agent of diseases.

The 5,10-methylenetetrahydrofolate reductase (*MTHFR*) acts as a key enzyme for regulating plasma total homocysteine levels, and is involved in the folate-dependent remethylation of homocysteine to methionine. *C677T* polymorphism in the *MTHFR* gene would be a potential risk factor for vascular disease [4]. The role of *C677T/MTHFR* in cardiovascular [5] and cerebrovascular [6] disease has been particularly emphasized. However, the results remain controversial, such as in the study conducted by Spence et al. [7], it was demonstrated that plasma Hcy, and not the *MTHFR* genotype, is significantly associated with carotid atherosclerosis. Based on the above analysis, the effect of *C677T/MTHFR* on CAS was investigated in Chinese Han adults. In addition, it was determined whether there was a correlation among *MTHFR* polymorphism, homocysteine level, and increased risk of CAS in the Chinese Han population, which might differ from other ethnics.

## Methods

### Participants

A total of 817 Chinese adults (Han), who voluntarily participated in a risk screening program for cardiovascular and cerebrovascular diseases and provided an informed consent, were randomly recruited from The First Affiliated Hospital of Chongqing Medical University between June 2016 and October 2017. Exclusion criteria: (1) subjects who were non-Han; (2) subjects with a history of stroke, Alzheimer’s-type dementia, intracranial infection, sinus thrombosis, demyelinating disease, Parkinson’s disease, and severe anxiety and/or depression; (3) subjects with a history of organic heart diseases (such as rheumatic heart disease, dilated cardiomyopathy, cor pulmonale, etc.); (4) subjects with a history of severe liver disease or renal insufficiency, thyroid disease (hyper- or hypothyroidism), autoimmune disease, or malignancy; (5) subjects with a history of a serious infection, major surgery, or trauma within the first four weeks of inclusion; (6) subjects with a history of lipid-lowering drugs, B vitamins and/or folic acid supplements within the first four weeks of inclusion; (7) pregnant subjects; (8) subjects with erroneous data. A total of 730 subjects were enrolled into the final statistical analysis. This research was approved by the Ethics Committee of The First Affiliated Hospital of Chongqing Medical University. Written informed consent was obtained from all subjects.

### Measurements

Data on demographic information (age and gender), disease history (hypertension and diabetes mellitus (DM)), and smoking history were collected through face-to-face interview with a structured questionnaire. Anthropometric indicators (height, weight and body mass index (BMI); BMI = weight / height^2^ [kg/m^2^]) were classified, as follows: < 25 kg/m^2^ (normal), ≥25 kg/m^2^ (overweight), and ≥ 30 kg/m^2^ (obese). Blood pressure: This was measured using an electronic sphygmomanometer. The subjects were instructed to rest for at least 10 min before the measurement, and the blood pressure is measured using the average of two systolic blood pressure (SBP) and diastolic blood pressure (DBP) measurements (the record interval was greater than three minutes). Hypertension [8]: Blood pressure was measured three times on different days within two weeks; SBP ≥140 mmHg and/or DBP ≥90 mmHg, or presently taking antihypertensive drugs.

The blood samples of subjects were collected after 12 h of fasting, and placed in EDTA anticoagulated tubes. Fasting total plasma homocysteine levels were determined by high performance liquid chromatography. An OLYMPUS AU5400 clinical biochemical analyzer was used to measure for triglycerides (TG), total cholesterol (TC), low density lipoprotein cholesterol (LDL-C), high-density lipoprotein cholesterol (HDL-C), and fasting blood glucose (FBG). TC ≥5.18 mmol/L was defined as hypercholesterolemia, TG ≥1.70 mmol/L was defined as high, LDL-C ≥ 3.37 mmol/L was defined as high, and HDL-C < 1.04 mmol/L was defined as low [9]. DM [10]: Three fasting blood glucose levels were measured on different days in two weeks; ≥7.0 mmol/L or oral glucose tolerance test (OGTT) two-hour plasma glucose > 11.1 mmol/L, or presently taking antidiabetic drugs. Hcy concentration ≥ 15 μmol/L was defined as HHcy [11].

All interviews were conducted by nurses or researchers, who previously participated in multiple trainings, and were blinded to participants’ *MTHFR* genotypes. All tests were conducted in accordance with established guidelines and procedures.

### Ultrasound measurement

The atherosclerosis index of the IMT was measured using a LOGIQ P5 color Doppler ultrasound system, equipped with a 10 L linear array broadband probe with 5–10 MHz. Subjects were instructed to maintain the supine and head-tilt position. The regions were scanned from 30 mm proximal to the beginning of the dilation of the bifurcation bulb to 15 mm distal to the flow divider of both common carotid arteries (CCAs). All measurements were conducted while scanning with the electronic caliper and recorded on photocopies. On a longitudinal scan of the CCAs at a point 10 mm proximal to the beginning of the dilation of each carotid artery bulb, IMT was measured. The mean of the IMT for the proximal and distal walls at the point of measurement was used to define IMT, and the inner-media thickness was taken as the mean of three measurements. Evaluation criteria: IMT < 1.0 mm for IMT normal, IMT = 1.0–1.5 mm for thickening, IMT ≥ 1.5 mm and local bulge thickening to the pelvic protrusion for carotid plaque formation. According to the carotid ultrasonography results, those subjects were divided into two groups: CAS group (IMT ≥ 1.0 mm) and normal group (IMT < 1.0 mm).

All the measurements were conducted by the same ultrasound doctor who had been engaged in ultrasound work for more than 5 years and had received unified training. A single reader interpreted all images. The inspection results were recorded in detail after checking. Both the sonographer and the reader were blinded to participants’ *MTHFR* genotypes.

### Determination of the *MTHFR* genotype

1. DNA extraction: After taking the EDTA anticoagulated peripheral blood of 200 μl, the genomic DNA was extracted using the whole blood genomic DNA rapid extraction kit (Tiangen Bio, Batch number: Q5502), and its purity and concentration were determined. Then the concentration was adjusted to 4 ng/μl.
2. Primer design: Primer 5.0 software was used to design the primer: Upstream primer: CATCCCTATTGGCAGGTTAC; downstream primer: GACGGTGCGGTGAGAGTG; synthesized by Shanghai Bioengineering Co. Reaction system: 5 μl of PCR master mix, 1.5 μl of the primer (0.8 μm), 2.5 μl of DdH2O, 1 μl of genomic DNA, and a total volume of 10 μl (The PCR master mix was purchased from Applied Biosystems, Product number: 4458687). Amplification conditions: Initial denaturation at 95 °C for 10 min; followed by 35 cycles of 96 °C for 30 s, 62 °C for 15 s, and 68 °C for 30 s; lastly, 72 °C for two minutes. Primer specificity was verified by PCR product electrophoresis and Sanger sequencing.
3. Product purification and PCR sequencing: 2 μl of direct sequencing master mix and 1 μl of reverse primer were added to the PCR amplification products. Reaction conditions: Enzymolysis at 37 °C for 15 min and denaturation at 80 °C for two minutes, followed by 25 cycles of 96 °C for one minute, and 96 °C for 10 s, 50 °C for five seconds, 60 °C for 75 s, and lastly, heat preservation at 4 °C.
4. Purification of sequencing products: BigDye XTerminator purification reagent SAM solution (45 μl) and XTerminator solution (10 μl) (purchased from Applied Biosystems Corporation, Article number: 4376484) were used, and shaking was performed for more than 10 s. Then, the SAM/XTerminator mixture (55 μl, uniform mixture) was placed into a 96-well plate. The PCR sequencing product was transferred to the reaction well using a medium pipette tip, fixed in an IKA MS3 basic shaker at 2,200 rpm, and shaken for 30 min. Then, the cells were centrifuged at 2,500 rpm for three minutes, and analyzed using a 3500 DX Genetic Analyzer.

The original sequence of 730 samples were all further aligned by NCBI-BLAST (*https://blast.ncbi.nlm.nih.gov/Blast.cgi*) to determine the similarity with *MTHFR* gene, and were divided into three types: *CC, CT* heterozygous and *TT* homozygous. All experiments were repeated three times.

### Statistical analysis

The SPSS 21.0 software was used for all statistical analyses. Continuous variables that were normally distributed were expressed as mean ± standard deviation (SD). Independent-sample t-test was used to detect the differences between CAS-group and control group. Median (interquartile range, IQR) was used to present skewed parameters, and Mann-Whitney U-test was used to detect the differences between CAS-group and control group. Discrete variables were expressed in percentage. Pearson’s chi-square test was used to determine whether there was a between-groups difference in allele or genotypic frequencies between CAS-group and control group. Associations of C677T/MTHFR with CAS were investigated through logistic regression analysis considering potential confounding risk variables, including age, gender, drinking, smoking, dietary habit, sleep quality, BMI, hypertension, dyslipidemia and DM, and the associations of C677T/MTHFR with CAS were also determined by sex through logistic regression analysis, adjusting all the potential confounding risk variables above. A dominant (*TT + CT* versus *CC*) and a recessive (*TT* versus *CT + CC*) model were used to examine the effect of the genotype. For multivariate risk predictors, the adjusted odds ratios (ORs) were given with the 95% confidence intervals (CIs). All tests were two-tailed, and *P* < 0.05 was used as the significance level.

## Results

### Basic characteristics of the subjects

The demographic and clinical data of CAS and non-CAS are presented in Table 1. A total of 266 subjects with CAS and 464 non-CAS enrolled in the present study. Age, hypertension, DM, SBP, DBP, FBG, TC, LDL-C and total plasma Hcy levels were significantly higher in the subjects with CAS, when compared to the control group.

**Table 1:** Clinical and Basic Characteristics in the general study populations

	CAS	Normal	Z-value	x^2^-value	*P*-value
N	266	464			
Age(y)	53(48,62)	48(42,53)	−8.761		<0.001^*^
Gender				0.000	0.997
male(n,%)	188(70.7)	328(70.7)			
female(n,%)	78(29.3)	136(29.3)			
Hypertension(n,%)	138(51.9)	149(32.1)		27.691	<0.001^#^
Diabetes(n,%)	52(19.5)	54(11.6)		8.525	0.004^#^
Smoking(n,%)	99(37.2)	184(39.7)		0.423	0.515
BMI (kg/m2)	24.76(22.51,26.48)	24.80(22.31,27.05)	−0.549		0.583
SBP (mmHg)	133(120,147)	126(114,137)	−4.759		<0.001^*^
DBP (mmHg)	81(73,90)	80(71,87)	−2.224		0.026^*^
hsCRP (mg/L)	1.52(0.73,1.73)	1.45(0.60,1.68)	−1.580		0.114
FBG (mmol/L)	5.6(5.1,6.4)	5.3(5.0,5.8)	−4.255		<0.001^*^
TC (mmol/L)	5.10(4.47,5.84)	4.78(4.28,5.46)	−3.729		<0.001^*^
TG (mmol/L)	1.60(1.15,2.40)	1.50(1.11,2.33)	−0.839		0.402
LDL (mmol/L)	3.32(2.74,3.92)	3.07(2.64,3.64)	−3.485		<0.001^*^
HDL (mmol/L)	1.34(1.12,1.59)	1.33(1.14,1.56)	−0.013		0.990
Hcy (mmol/L)	10.40(8.62,13.50)	9.30(7.70,11.60)	−4.713		<0.001^*^

*BMI* body mass index, *SBP* systolic blood pressure, *DBP* diastolic blood pressure, *TG* triglycerides, *TC* total cholesterol, *LDL-C* low density lipoprotein cholesterol, *HDL-C* high-density lipoprotein cholesterol, *FBG* fasting blood glucose, Diabetes: fasting plasma glucose ≥7.0 mmol/L or OGTT 2 h plasma glucose > 11.1 mmol/L, or currently taking antidiabetic drugs

Values are mean ± standard (SD), median (interquartile range, IQR) or percentage

^*^p<0.05 between cases and controls by Mann-Whitney U-test;^#^*p*<0.05 between cases and controls by *X*^*2*^ test

### The correlation of HHcy and the *MTHFR* genotype

The logistic regression analysis revealed that subjects who carried the homozygous *T/T* (adjusted OR = 11.66, 95% CI: 5.96–22.83, *P* < 0.001) had a higher risk of HHcy. However, there were no similar significant associations observed in the subjects who carried the heterozygotes *C/T* and homozygous *C/C* (Table 2).

**Table 2:** Risk factors of hyperhomocysteinemia by Binary logistic analysis

	B-value	SE	Wald	*P-value*	OR	95%CI
Age	1.196	0.304	15.52	< 0.001^*^	3.31	1.82 ~ 6.00
Gender	1.445	0.434	11.059	0.001^*^	4.24	1.81 ~ 9.94
Smoking	0.320	0.273	1.371	0.242	1.38	0.81 ~ 2.35
Drinking	0.089	0.287	0.097	0.755	1.09	0.62 ~ 1.92
Dietary habit	−0.078	0.264	0.086	0.769	0.93	0.55 ~ 1.55
Hypertension	0.461	0.269	2.938	0.087	1.59	0.94 ~ 2.69
FBG						
< 5.5 mmol/L			2.811	0.245		
5.5–7 mmol/L	−0.002	0.284	0.000	0.995	1.00	0.57 ~ 1.74
≧7 mmol/L	−0.684	0.426	2.573	0.109	0.51	0.22 ~ 1.16
MTHFR C677T						
CC			68.307	< 0.001^*^		
CT	0.189	0.321	0.348	0.555	1.21	0.65 ~ 2.27
TT	2.456	0.343	51.366	< 0.001^*^	11.66	5.96 ~ 22.83

Age(y): <60 = 0,≥60 = 1; Gender: Female = 0,Male = 1; Dietary habit: low-salt and/or low-fat =0, high-salt and/or high-fat = 1; Smoking:

No =0, yes = 1.Drinking: No = 0, Yes = 1. Hypertension: normal = 0, Hypertension = 1. ^*^P<0.05 was considered to be statistically significant

### Association of HHcy with the *MTHFR* genotype

When individuals were classified according to *MTHFR* genotype, ANOVA was performed to determine the difference in the prevalence of HHcy among the different groups. As presented in Table 3, subjects with the *T/T* homozygote had a higher prevalence of HHcy, when compared to the subjects with the *C/C* or *C/T* genotype, and there was a statistically significant difference (*P* < 0.001). The same results were found in the stratified analysis by gender.

**Table 3:** Comparison of incidence of hyperhomocysteinemia among different MTHFR genotypes

	MTHFR Genotype			*X*^2^*P*
	C/C	C/T	T/T	
ALL(*n* = 86)				
HHcy				
n	18	30	38	
%	5.9	8.9	43.7	< 0.001^*^
Female(*n* = 9)				
HHcy				
n	2	4	3	
%	1.7	5.3	15.0	0.023^*^
Male(*n* = 77)				
HHcy				
n	16	26	35	
%	8.5	10.0	52.2	< 0.001^*^

*HHcy* hyperhomocysteinemia

^*^P<0.05 was considered to be statistically significant

### *MTHFR* allele and genotype frequencies

The results of the single nucleotide polymorphism association analyses are presented in Table 4. There were 307 *MTHFR 677C/C* homozygotes (42.1%), 336*677C/T* heterozygotes (46.0%), and 87,*677 T/T* homozygotes (11.9%), and 950 MTHFR allele *C* and 510 *T* allele. The *MTHFR C677T* was found to be in Hardy-Weinberg equilibrium (HWE) in the subjects (*P* > 0.05 by × ^2^). In the *MTHFR C677T*, there were significant differences in *T* allele (OR = 2.31, 95% CI: 1.85–2.88, *P* < 0.001), *C/T* genotype (OR = 4.14, 95% CI: 2.90–5.92, *P* < 0.001) and *T/T* genotype (OR = 4.20, 95% CI: 2.52–6.97, *P* < 0.001) between CAS-group and control group. The same results were found in the stratified analysis by gender.

**Table 4:** Allele and genotype frequencies of polymorphisms in MTHFR between CAS group and control group

	Allele/Genotype	CAS-group		Control group		OR (CI 95%)	*P*-value	*P* for HWE
		(*n* = 266)		(*n* = 464)				
		n	%	n	%			
ALL	C	281	52.8	669	72.1	1		0.74
	T	251	47.2	259	27.9	2.31(1.85,2.88)	P < 0.001^*^	
	C/C	58	21.8	249	53.7	1		
	C/T	165	62	171	36.9	4.14 (2.90,5.92)	P < 0.001^*^	
	T/T	43	16.2	44	9.5	4.20(2.52,6.97)	P < 0.001^*^	
Women	C	82	52.6	231	84.9	1		
	T	74	47.4	41	15.1	5.08(3.22,8.03)	P < 0.001^*^	
	CC	16	20.5	103	75.7	1		
	CT	50	64.1	25	18.4	12.88(6.31,26.26)	P < 0.001^*^	
	TT	12	15.4	8	5.9	9.66(3.42,27.27)	P < 0.001^*^	
Men	C	199	52.9	438	66.8	1		
	T	177	47.1	218	33.2	1.79(1.38,2.32)	P < 0.001^*^	
	CC	42	22.3	146	44.5	1		
	CT	115	61.2	146	44.5	2.74(1.80,4.17)	P < 0.001^*^	
	TT	31	16.5	36	11	2.99(1.67,5.40)	P < 0.001^*^	

*MTHFR* Methylenetetrahydrofolate reductase, *OR* Odds ratio, *C*I Confidence interval, *HWE* Hardy-Weinberg equilibrium

^*^P<0.05 was considered to be statistically significant

### Carotid atherosclerosis, *C677T/MTHFR* and CAS-related risk factors

There was no significant difference in age between gene groups (x^2^ = 1.09, df = 2, *P* = 0.99). The logistic regression analysis revealed that the subjects who carried the heterozygous *C/T* (adjusted OR = 4.06, 95% CI: 2.76–5.98, *P* < 0.001) or homozygous *T/T* (adjusted OR = 3.14, 95% CI: 1.73–5.69, *P* < 0.001) genotypes had a significantly greater risk of CAS (Table 5). Furthermore, the subjects with Hcy ≥15 μmol/L also had a higher risk of CAS (adjusted OR = 2.01, 95% CI: 1.14–3.57, *P* < 0.017; Table 5).

**Table 5:** Result of the risk factors of Carotid Atherosclerosis by Logistic regression analysis

	B-value	SE	Wald	P-value	OR	95%CI
Age	0.895	0.229	15.336	< 0.001^*^	2.45	1.56 ~ 3.83
Gender	−0.507	0.238	4.532	0.033^*^	0.60	0.38 ~ 0.96
Smoking	−0.070	0.202	0.120	0.729	0.93	0.63 ~ 1.38
Drinking	0.639	0.200	10.162	0.001^*^	1.89	1.28 ~ 2.81
Dietary habit	−0.371	0.202	3.378	0.066	0.69	0.47 ~ 1.03
Sleep quality						
poor			2.667	0.264		
aduquate	0.389	0.259	2.248	0.134	1.48	0.89 ~ 2.45
good	0.455	0.301	2.283	0.131	1.58	0.87 ~ 2.85
BMI	−0.240	0.210	1.313	0.252	0.79	0.52 ~ 1.19
Hypertension	0.748	0.196	14.609	< 0.001^*^	2.11	1.44 ~ 3.10
FBG						
< 5.5 mmol/L			11.022	0.004^*^		
5.5–7 mmol/L	0.553	0.197	7.862	0.005^*^	1.74	1.18 ~ 2.56
≧7 mmol/L	0.749	0.291	6.612	0.010^*^	2.12	1.20 ~ 3.75
hsCRP(Q1)			3.079	0.380		
hsCRP = 1	0.264	0.252	1.093	0.296	1.30	0.79 ~ 2.13
hsCRP = 2	0.444	0.258	2.950	0.086	1.56	0.94 ~ 2.59
hsCRP = 3	0.299	0.258	1.340	0.247	1.35	0.81 ~ 2.24
TC	0.334	0.276	1.461	0.227	1.40	0.81 ~ 2.40
TG	−0.195	0.193	1.023	0.312	0.82	0.56 ~ 1.20
LDL-C	0.458	0.278	2.715	0.099	1.58	0.92 ~ 2.72
HDL-C	−0.391	0.268	2.123	0.145	0.68	0.40 ~ 1.14
Hcy	0.700	0.292	5.746	0.017^*^	2.01	1.14 ~ 3.57
MTHFR C677T						
CC			51.512	< 0.001^*^		
CT	1.401	0.198	50.270	< 0.001^*^	4.06	2.76 ~ 5.98
TT	1.143	0.304	14.165	< 0.001^*^	3.14	1.73 ~ 5.69

*BMI* body mass index, *TG* triglycerides, *TC* total cholesterol, *LDL-C* low density lipoprotein cholesterol, *HDL-C* high-density lipoprotein cholesterol, *hsCRP* high-sensitivity C-reative protein, Hcy: Homocysteine; Hypertension: normal = 0, Hypertension = 1; Hcy: <15 mmol/L = 0, ≥15 mmol/L = 1; TC:<5.18 mmol/L = 0, ≥5.18 mmol/L = 1;TG: <1.70 mmol/L = 0,≥1.70 mmol/L = 1;LDL-C: <3.37 mmol/L = 0, ≥3.37 mmol/L = 1; HDL-C: ≥1.04 mmol/L = 0,< 1.04 mmol/L = 1); HCY: <15 μmol / L = 0,≥ 15 μmol / L = 1; hsCRP (Quadripartite grouping):the first quartile = 0, the second quartile =1, the third quartile =2, the highest quartile =3; Gender: Female = 0,Male = 1; Smoking:No =0, yes = 1.Drinking: No = 0, Yes = 1

^*^P<0.05 was considered to be statistically significant

In the model 1 *(CT + TT* vs *CC), C677T/MTHFR* was significantly associated with the prevalence of CAS. All the adjusted OR for CAS was 3.87 (95% CI, 2.67 to 5.62) in all, following adjustment for age, gender, drinking, smoking, dietary habit, sleep quality, BMI, hypertension, FBG, hsCRP, TC, TG, LDL-C, HDL-C and Hcy. There was no significant difference in age between gene groups (*P* = 0.90). The same results were observed in the stratified analysis by gender, and all adjusted OR values for CAS were 17.18 (95% CI, 7.27 to 40.49) in women and 2.57 (95% CI, 1.65 to 3.99) in men, following adjustment for age, drinking, smoking, dietary habit, sleep quality, BMI, hypertension, FBG, hsCRP, TC, TG, LDL-C, HDL-C and Hcy. There was no significant difference in age between gene groups, either in man or women(*P* = 0.13, 0.80, respectively). In the model 2 *(CC + CT* versus *TT), C677T/MTHFR* was significantly associated with the prevalence of CAS (Table 6).

**Table 6:** ORs of presence of carotid atherosclerosis by *C677T/MTHFR*

	model 1		model2	
	CC	CT + TT	CC + CT	TT
ALL(N = 730)				
CAS,%	21.8	51.8	37.2	54.0
All adjusted OR^a^	1	3.87(2.67–5.62) ^*^	1	1.42(0.82–2.45)
Women(*n* = 214)				
CAS,%	13.4	65.3	34.0	60.0
All adjusted OR^a^	1	17.18(7.27–40.49) ^*^	1	2.06(0.67–6.38)
Men(*n* = 516)				
CAS,%	27.1	47.9	38.5	52.2
All adjusted OR^a^	1	2.57(1.65–3.99) ^*^	1	1.37(0.71–2.63)

^a^ logistic regression analysis; CAS, Carotid Atherosclerosis; adjusted for age, gender, drinking, smoking, Dietary habit, Sleep quality, BMI, Hypertension, FBG, hsCRP, TC, TG, LDL-C, HDL-C, Hcy. ^*^P<0.05 was considered to be statistically significant

## Discussion

In the present study, it was found that the *C/T* heterozygote and *T/T* homozygote carriers of the *MTHFR C677T* gene were more prone to having CAS, when compared to the *C/C* homozygote carriers, and in the model *(CC* versus *CT + TT), C677T/MTHFR* was significantly associated with the prevalence of CAS (OR = 3.87, 95% CI: 2.67–5.62) in all, the same results were observed in the stratified analysis by gender, and all adjusted OR values for CAS were 17.18 (95% CI, 7.27 to 40.49) in women and 2.57 (95% CI, 1.65 to 3.99) in men, intimating that the *T* allele was the predisposing gene for CAS, and that the elevated Hcy levels was probably attributed, which promote atherosclerosis.

Rapid demographic aging is becoming a vigilant public health issue. Age-related chronic non-communicable diseases, such as CAS, have gradually increased. Atherosclerosis is the result of multifactorial interactions, which are affected by environmental and genetic factors [12, 13]. Previous studies have revealed that the *T/T* genotype of *C677T/MTHFR* is correlated with greater risk of cardiovascular disease [4, 14]. A study with a sample size of 3247 subjects (30 to 89 years of age; 1693 women, 1554 men) showed that the *MTHFR T/T* genotype is a risk factor for carotid stenosis in a Japanese General Population [15] . Kawamoto, R.et al. [16] showed the presence of a T allele was a significant risk factor for IMT thickening and further supported the role of *C677T/MTHFR* in common carotid atherosclerosis. However, the conclusions still remain controversial. Pramukarso et al. [17] suggested that the *MTHFR* gene *C677T* mutation leads to an increase in Hcy. However, there was no significant correlation with CAS. Small sample size, race diversity and the inadequate adjustment of confounding factors, such as smoking, drinking, sleeping quality and dietary habit, would attribute to these inconsistencies. Therefore, *MTHFR* variant *C677T* is a relatively independent risk factor for CAS, or indirectly promotes atherosclerosis by increasing plasma Hcy levels, which has become a hot topic in recent years. Thus, given this potential reason, the effect of *C677T/ MTHFR* on CAS was examined in Chinese Han populations with a more adequate adjustment of lifestyle (smoking, drinking, dietary habit, sleep quality), and relevant basic characteristics (age, gender, BMI), hypertension, FBG, hsCRP, TC, TG, LDL-C, HDL-C and Hcy.

In the present study, it was found that a mutation in the *MTHFR* gene is a risk factor of CAS in the Chinese Han population, and that plasma total Hcy has a significant association with CAS. However, the mechanisms behind CAS are far from being understood, and need to be further investigated. The following potential mechanisms might explain the contribution of the *C677T/MTHFR* polymorphisms to CAS predisposition: (1) *MTHFR*, which is a key enzyme in methionine and folate metabolism, influences DNA metabolism and maintains the proper Hcy levels in vivo. Mutations in the *C677T/MTHFR* gene leads to a starvation or activity decrease of *MTHFR* (subjects who carry the *C/T* heterozygous genotype polymorphism only have 70% of normal enzyme activity, while subjects who carry the *T/T* homozygous genotype only have 30% of normal enzyme activity [18]), which impeding the conversion of Hcy to methionine, causes a decrease in serum folate level, an increase in Hcy level, and DNA hypomethylation [19–21]. (2) Hcy plays an important role in endothelial cells damage, accelerating atherosclerosis onset and progress, and contributes to the formation of unstable plaque through inflammatory factors, oxidative stress, endoplasmic reticulum stress and immune responses [22, 23]. Furthermore, Hcy leads to endothelial cells injury and the oxidation of lipids by damaging the nitric oxide system, and also oxidizes low-density lipoproteins, and accelerates the atherosclerosis process [24, 25]. Contrary to prior studies, there were literatures have revealed that the *T/T* genotype was significantly correlated with elevated plasma total Hcy levels only in folate deficient subjects [26–28]. That is, subjects with the *T/T* genotype and received sufficient folate would not have an increased risk of cardiovascular disease via HHcy. Thus, the population with a history of folic acid supplements within the first four weeks before the trial was excluded. (3) As an indirect methyl donor, *MTHFR* participates in the methylation processes of DNA, RNA and proteins (purine and thymidine syntheses). These result in a series of vascular diseases.

Simultaneously, it was detected that the mutations in the *C677T/MTHFR* gene leads to the increase in Hcy level, and the possible potential mechanisms have been mentioned above. On the other hand, it was also found that the risk of HHcy was greater in the male gender, when compared to the female gender (OR = 4.24, 95% CI: 1.18–9.94, *P* = 0.001). The most likely explanation for this observation was that plasma total Hcy levels are significantly associated with testosterone, and inversely correlated with estradiol [29, 30].

However, this was diverse with a previous study, [15] and under stratification by gender, no differences were observed in the association between the *C/T* heterozygote and *T/T* homozygotes genotype, and CAS. The potential reason was that the sample size used to detect the gene and environmental interactions was small. Hence, there is a need for a substantially larger sample size, in order to detect the genetic or environmental effects alone [31, 32]. Furthermore, the present results revealed that mutations in the *C677T/MTHFR* gene may be a contributing factor to the higher risk profile for the development of arteriosclerosis, which were also supported by previous studies [17, 33, 34].

There are several limitations in the present study. There was a lack of dietary information in the data of the study subjects; and there was a lack in the data of the *MTHFR* serum activity or folate serum levels. In clarifying some of the apparent discrepancies in the relationship among *C677T/MTHFR* genotype, plasma total homocysteine levels and CAS, the knowledge of these might have been a contributing factor, Further study is needed. Nonetheless, the present data are consistent with those in previous studies, in which the *MTHFR* gene *C677T* mutation is statistically associated with HHcy and CAS.

## Conclusion

In conclusion, it was found that mutations in the *C677T/MTHFR* gene are risk factors for CAS in the Chinese Han population. The *T* allele may be a susceptibility gene of carotid plaques. Exploring the potential association of *MTHFR* SNPs with CAS susceptibility may be conducive to personalized diagnosis, which can lead to both disease prevention and modification.

## Data Availability

The datasets used and analyzed during the current study are available from the corresponding author on reasonable request.

## Abbreviations

*MTHFR*: Methylenetetrahydrofolate reductase
CAS: Carotid atherosclerosis
IMT: Intima-media thickness
Hcy: Homocysteine
HHcy: Hyperhomocysteinemia
CVD: Cardiovascular disease
DM: Diabetes mellitus
BMI: Body mass index
SBP: Systolic blood pressure
DBP: Diastolic blood pressure
TG: Triglycerides
TC: Total cholesterol
LDL-C: Low density lipoprotein cholesterol
HDL-C: High-density lipoprotein cholesterol
FBG: Fasting blood glucose
OGTT: Oral glucose tolerance test
CCAs: Common carotid arteries
IQR: Interquartile range
HWE: Hardy-Weinberg equilibrium
ORs: Odds ratios
CIs: Confidence intervals

## References

[1] Albert SM (2015). Insights on the early-life origins of Alzheimer's disease: relevance for primary prevention?. Neuroepidemiology.

[2] Skovierova H, Vidomanova E, Mahmood S, Sopkova J, Drgova A, Cervenova T, Halasova E, Lehotsky J. The Molecular and Cellular Effect of Homocysteine Metabolism Imbalance on Human Health. Int J Mol Sci. 2016;17(10):1733. 10.3390/ijms17101733 PMC5085763 27775595

[3] Werstuck GH, Lentz SR, Dayal S, Hossain GS, Sood SK, Shi YY, Zhou J, Maeda N, Krisans SK, Malinow MR (2001). Homocysteine-induced endoplasmic reticulum stress causes dysregulation of the cholesterol and triglyceride biosynthetic pathways. J Clin Investig.

[4] Frosst P, Blom HJ, Milos R, Goyette P, Sheppard CA, Matthews RG, Boers GJ, den Heijer M, Kluijtmans LA, van den Heuvel LP (1995). A candidate genetic risk factor for vascular disease: a common mutation in methylenetetrahydrofolate reductase. Nat Genet.

[5] Bennouar N, Allami A, Azeddoug H, Bendris A, Laraqui A, El Jaffali A, El Kadiri N, Benzidia R, Benomar A, Fellat S (2007). Thermolabile methylenetetrahydrofolate reductase C677T polymorphism and homocysteine are risk factors for coronary artery disease in Moroccan population. J Biomed Biotechnol.

[6] Morita H, Kurihara H, Tsubaki S, Sugiyama T, Hamada C, Kurihara Y, Shindo T, Oh-Hashi Y, Kitamura K, Yazaki Y (1998). Methylenetetrahydrofolate reductase gene polymorphism and ischemic stroke in Japanese. Arterioscler Thromb Vasc Biol.

[7] Spence JD, Malinow MR, Barnett PA, Marian AJ, Freeman D, Hegele RA (1999). Plasma homocyst(e) ine concentration, but not MTHFR genotype, is associated with variation in carotid plaque area. Stroke.

[8] China. CoC-C-VDoGSo, Association CCoCPoCMD (2017). Chinese expert consensus on the diagnosis and treatment of hypertension in the elderly(2017). Chin J Intern Med.

[9] Dyslipidemia JCftRotGfPaTo (2016). Guidelines for the prevention and treatment of dyslipidemia in Chinese adults (revised 2016). Chin Circ J.

[10] Sacks DB, Bruns DE, Goldstein DE, Maclaren NK, Mcdonald JM, Marian P (2002). Guidelines and recommendations for laboratory analysis in the diagnosis and management of diabetes mellitus. Clin Chem.

[11] Malinow MR, Bostom AG, Krauss RM (1999). Homocyst(e) ine, diet, and cardiovascular diseases: a statement for healthcare professionals from the nutrition committee, American Heart Association. Circulation.

[12] Hu EA, Steffen LM, Grams ME, Crews DC, Coresh J, Appel LJ, Rebholz CM. Dietary patterns and risk of incident chronic kidney disease: the Atherosclerosis Risk in Communities study. Am J Clin Nutr. 2019;110(3):713–21. 10.1093/ajcn/nqz146 PMC6736122 31386145

[13] Lechner K, von Schacky C, McKenzie AL, Worm N, Nixdorff U, Lechner B, Krankel N, Halle M, Krauss RM, Scherr J. Lifestyle factors and high-risk atherosclerosis: Pathways and mechanisms beyond traditional risk factors. Eur J Prev Cardiol. 2020;27(4):394-406. 10.1177/2047487319869400 PMC7065445 31408370

[14] Clarke R, Daly L, Robinson K, Naughten E, Cahalane S, Fowler B, Graham I (1991). Hyperhomocysteinemia: an independent risk factor for vascular disease. N Engl J Med.

[15] Nozomu I, Tomohiro K, Yoshihiro K, Toshifumi M, Takashi A, Shunroku B, Jun O, Hitonobu T, Toshio O (2003). Association of methylenetetrahydrofolate reductase gene polymorphism with carotid atherosclerosis depending on smoking status in a Japanese general population. Stroke.

[16] Kawamoto R, Kohara K, Tabara Y, Miki T, Doi T, Tokunaga H, Konishi I (2001). An association of 5,10-methylenetetrahydrofolate reductase (MTHFR) gene polymorphism and common carotid atherosclerosis. J Hum Genet.

[17] Pramukarso DT, Faradz SM, Sari S, Hadisaputro S (2015). Association between methylenetetrahydrofolate reductase (MTHFR) polymorphism and carotid intima medial thickness progression in post ischaemic stroke patient. Ann Transl Med.

[18] Jain M, Pandey P, Tiwary NK, Jain S (2012). MTHFR C677T polymorphism is associated with hyperlipidemia in women with polycystic ovary syndrome. J Hum Reprod Sci.

[19] Hanson NQ, Aras O, Yang F, Tsai MY (2001). C677T and A1298C polymorphisms of the methylenetetrahydrofolate reductase gene: incidence and effect of combined genotypes on plasma fasting and post-methionine load homocysteine in vascular disease. Clin Chem.

[20] Maria Grazia A, Nicoletta B, Franca C, Debora B, Elisabetta A, Serena M, Samantha M, Maria Giovanna C, Andrea B, Aldo C (2003). Methylenetetrahydrofolate reductase gene C677T polymorphism, homocysteine, vitamin B12, and DNA damage in coronary artery disease. Hum Genet.

[21] Bharatkumar VP, Nagaraja D, Christopher R (2014). Hyperhomocysteinemia and methylenetetrahydrofolate reductase C677T polymorphism in cerebral veno-sinus thrombosis. Clin Appl Thromb Hemost.

[22] Yan-Yan L (2010). Relationship of serum homocysteine and high sensitivity C-reactive protein in elderly people with essential hypertension. Int J Clin Pract.

[23] Sethi AS, Lees DM, Douthwaite JA, Dawnay AB, Corder R (2006). Homocysteine-induced endothelin-1 release is dependent on hyperglycaemia and reactive oxygen species production in bovine aortic endothelial cells. J Vasc Res.

[24] Kloppenborg RP, Nederkoorn PJ, Graaf YVD, Geerlings MI (2011). Homocysteine and cerebral small vessel disease in patients with symptomatic atherosclerotic disease. The SMART-MR study. Atherosclerosis.

[25] Codoñer-Franch P, Tavárez-Alonso S, Murria-Estal R, Megías-Vericat J, Tortajada-Girbés M, Alonso-Iglesias E (2011). Nitric oxide production is increased in severely obese children and related to markers of oxidative stress and inflammation. Atherosclerosis.

[26] Ma J, Stampfer MJ, Hennekens CH, Frosst P, Selhub J, Horsford J, Malinow MR, Willett WC, Rozen R (1996). Methylenetetrahydrofolate reductase polymorphism, plasma folate, homocysteine, and risk of myocardial infarction in US physicians. Circulation.

[27] Harmon DL, Woodside JV, Yarnell JW, Mcmaster D, Young IS, Mccrum EE, Gey KF, Whitehead AS, Evans AE (1996). The common 'thermolabile' variant of methylene tetrahydrofolate reductase is a major determinant of mild hyperhomocysteinaemia. Qjm.

[28] Jacques PF, Bostom AG, Williams RR, Ellison RC, Eckfeldt JH, Rosenberg IH, Selhub J, Rozen R (1996). Relation between folate status, a common mutation in methylenetetrahydrofolate reductase, and plasma homocysteine concentrations. Circulation.

[29] Dierkes J, Jeckel A, Ambrosch A, Westphal S, Luley C, Boeing H (2001). Factors explaining the difference of total homocysteine between men and women in the European investigation into Cancer and nutrition Potsdam study. Metab Clin Exp.

[30] Malinow MR, Duell PB, Hess DL, Anderson PH, Kruger WD, Phillipson BE, Gluckman RA, Block PC, Upson BM (1998). Reduction of plasma homocyst(e) ine levels by breakfast cereal fortified with folic acid in patients with coronary heart disease. N Engl J Med.

[31] Efird JT, Hong MK (2008). Computing differential sample size for case-control studies of gene-environment interaction. Ethn Dis.

[32] Hamajima N, Mutoh H, Eguchi H, Honda H (2005). Minimal sizes of cases with a susceptible genotype and minimal odds ratios among susceptible individuals in case-control studies. Asian Pac J Cancer Prev.

[33] Senemar S, Saffari B, Sharifkazemi MB, Bahari M, Jooyan N, Dehaghani ED, Yavarian M (2013). 5,10-methylene tetrahydrofolate reductase C677T gene polymorphism, homocysteine concentration and the extent of premature coronary artery disease in southern Iran. EXCLI J.

[34] Abd El-Aziz TA, Mohamed RH (2017). Influence of MTHFR C677T gene polymorphism in the development of cardiovascular disease in Egyptian patients with rheumatoid arthritis. Gene.

